# Retention of nurses in rural Nepal: are short term contracts the answer?

**DOI:** 10.1186/1472-6963-14-S2-P80

**Published:** 2014-07-07

**Authors:** Joanna Morrison, Rita Thapa, Neha Batura, Jolene Skordis-Worrall

**Affiliations:** 1 Institute for Global Health, University Collge London, London, UK; 2 Nick Simons Institute, Kathmandu, Nepal

## Background

Shortages of health workers in rural areas are a global problem. The problem is more acute in poorer countries where the distribution of skilled, supported and motivated health workers is affected by internal and international migration. In Nepal, most women deliver at home, and the government has prioritized training and recruitment of nurses in rural areas to meet development goals.

Health sector reform for increased retention: Decentralisation of authority to district level health facility management committees (HFMCs) has the potential to increase public accountability and make health services more locally responsive. The Government of Nepal is encouraging local recruitment of nurses by HFMCs to increase access to maternal health care. Our research describes the differences between nurses on different contracts, examining their motivation, job satisfaction and retention in rural areas.

## Materials and methods

We used a mixed methods case study design, and sampled government District Hospitals, Health Posts and Primary Health Care Centres in three districts of Western Nepal with high and low numbers of locally contracted nurses. We interviewed nurses using the ‘Job Satisfaction Survey’ questionnaire. We also conducted qualitative in-depth interviews with nurses, and in-charges, and focus group discussions with HFMCs and women’s groups.

## Results

We found few staff nurses in post, and it was difficult to recruit this cadre in rural areas. We found that contracted nurses tended to be younger and less experienced. Many were from the local area or had some family connection nearby. HFMC contracted nurses had lower salaries, and worse terms and conditions than permanent nurses. They were usually expected to work 24 hours a day, seven days a week. They were motivated by the lack of employment opportunities and the need to maintain and develop their skills. Community members and in-charges felt that contract nurses were more motivated and worked harder than permanent nurses.

## Conclusions

In order to meet millennium goals for maternal and child health, it is essential to increase access to skilled birth attendants in rural areas. Although the strategy of local recruitment of nurses may be enabling 24 hour service provision, locally contracted nurses may find it difficult to deal with complicated deliveries without adequate support. Their lack of job security, and difficult terms and conditions may not foster longer-term retention in rural areas. We recommend that local recruitment of nurses should be part of a package of support to rural health facilities in order to increase retention.

